# Two Intriguing Cases of Stanford Type A Acute Aortic Dissection

**DOI:** 10.7759/cureus.6986

**Published:** 2020-02-13

**Authors:** Taha Ahmed, Raunak Nair, Hassan Lak, Samra Haroon Lodhi, Anjli Maroo

**Affiliations:** 1 Internal Medicine, Cleveland Clinic Foundation, Cleveland, USA; 2 Internal Medicine, Cleveland Clinic - Fairview Hospital, Cleveland, USA; 3 Internal Medicine, King Edward Medical University/Mayo Hospital, Lahore, PAK; 4 Cardiology, Cleveland Clinic Fairview Hospital, Cleveland, USA

**Keywords:** acute aortic dissection, transthoracic echocardiogram, pericardiocentesis, aortogram, pericardial effusion

## Abstract

Stanford type A acute aortic dissection (AAD) is a life-threatening illness that presents with chest pain and hemodynamic instability. Prompt and accurate evaluation and management are critical for survival as it is a cardiac surgical emergency. We aim to highlight the physicians about this potentially fatal condition, by reporting two cases of Stanford type A AAD, with atypical presentations that were initially misdiagnosed.

## Introduction

Acute aortic dissection (AAD) presents initially as a diverse array of signs and symptoms, making an early and accurate diagnosis arduous. The Stanford classification is divided into two groups, A and B, depending on whether the ascending aorta is involved. The Stanford type A AAD involves the ascending aorta and/or the aortic arch and has a higher mortality, requiring prompt surgical treatment [1]. The initial incidence of misdiagnosis is up to 40%, due to its clinical and epidemiologic overlap with acute coronary syndrome [2]. We describe two cases with different presentations found to have Stanford type A AAD, emphasizing that a high index of clinical suspicion should be kept in mind for this condition with any patient presenting with chest pain and associated risk factors [3,4].

## Case presentation

Case no. 1

**Figure 1:**
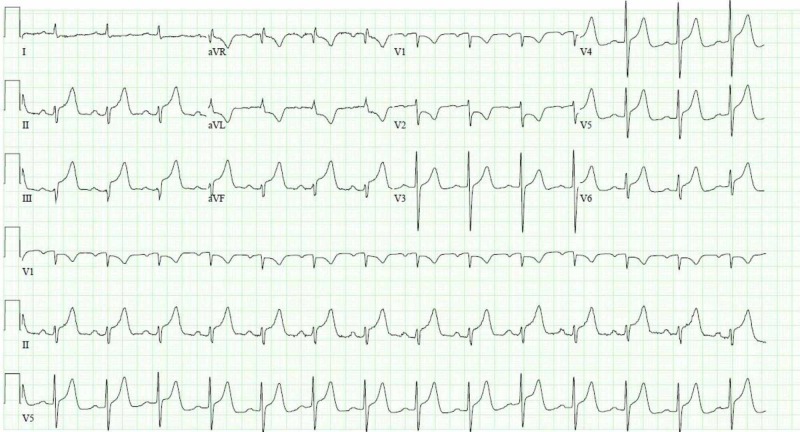
Electrocardiogram on presentation showing ST elevations in leads II, III, aVF, V4-6 with reciprocal ST depressions in leads I and aVL

A 66-year-old Asian male with a past medical history of uncontrolled hypertension presented to the emergency department with chest pain of one hour in duration. The pain was described as abrupt, pressure like, moderate with no alleviating or aggravating factors. The patient was in acute distress with an insignificant cardiorespiratory exam. Vitals on presentation revealed that he was normotensive (125/50 mmHg) with a heart rate of 70 beats per minute and afebrile. Electrocardiogram revealed ST-segment elevation in leads II, III, aVF, V4-6 with reciprocal ST depressions in V2 and aVL (Figure 1).

**Figure 2:**
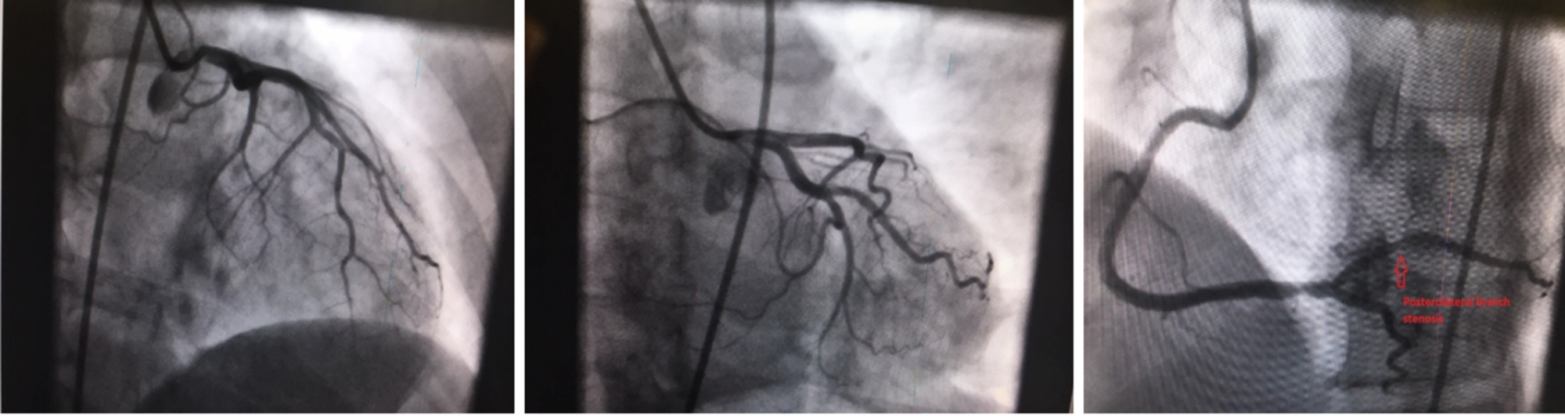
Left heart catheterization with normal left-sided coronary circulation and approximately 70% stenosis of the posterolateral branch of the right coronary artery

Complete blood count, metabolic panel, and initial troponin T were normal. The patient was administered aspirin, and was commenced on heparin infusion and immediately taken to the cardiac catheterization laboratory. Left heart catheterization showed no significant stenosis in the left-sided circulation; right coronary artery (RCA) was the dominant vessel with 70% stenosis of the posterolateral branch (Figure 2).

**Figure 3:**
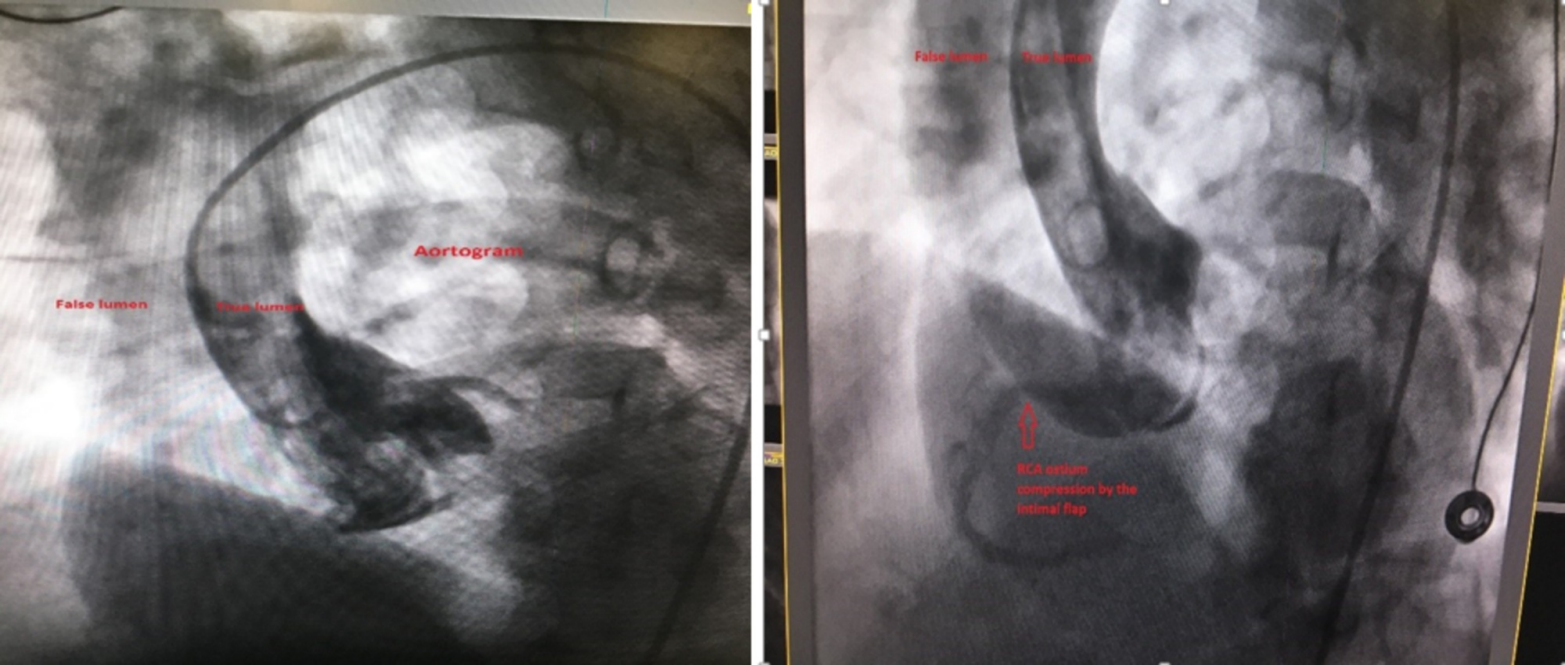
Aortogram revealing true and false lumens of the ascending aorta with an intimal flap intermittently compressing the ostia of the right coronary artery

With a presentation of acute inferoseptal myocardial infarction, the posterolateral branch was suspected to be the cause and a drug-eluting stent was placed. Prior to withdrawing the catheter, a left ventriculogram was performed and revealed a non-dilated left ventricle, severely dilated ascending aorta with evidence of an intimal flap. Subsequently, an aortogram revealed type A aortic dissection with severe aortic regurgitation. Furthermore, the flap intermittently compressed the ostium of the RCA (Figure 3).

The patient was started on intravenous bet-blockers and transferred for evaluation and surgical intervention for Stanford type A AAD.

Case no. 2

**Figure 4:**
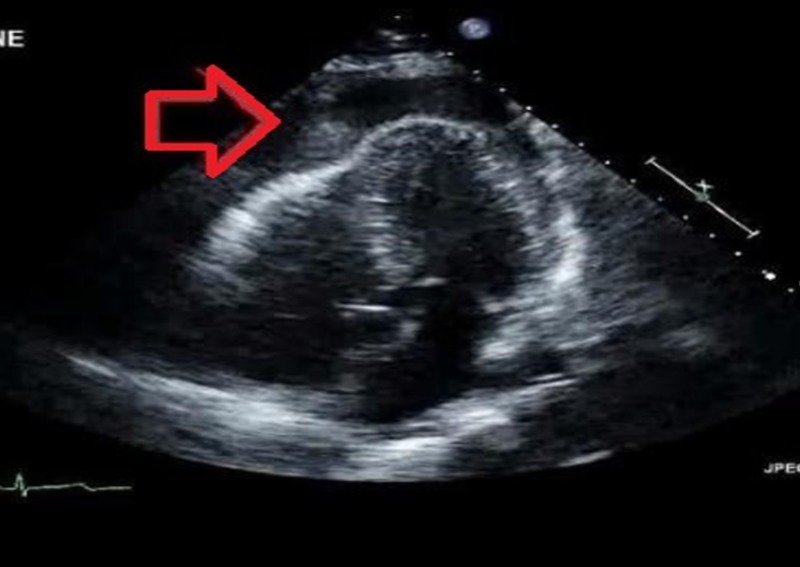
Transthoracic echocardiogram showing circumferential pericardial effusion

A 61-year-old female with a history of chronic alcoholism and uncontrolled hypertension was referred to our facility with a working diagnosis of acute coronary syndrome. She had an initial presentation of chest pain and dizziness for one day. The pain was sudden in onset, mid-epigastric, and worsened with lying flat. Systemic examination did not reveal any significant findings. Her vitals on presentation revealed she was hypotensive (94/62 mmHg), tachycardiac (hazard ratio, 111 beats per minute), and afebrile. Electrocardiogram showed trivial ST abnormalities. Laboratory findings were significant for an elevated anion gap of 27, acidosis with a HCO_3_ of 16, and creatinine of 1.6. Transthoracic echocardiogram revealed circumferential pericardial effusion (Figure 4).

**Figure 5:**
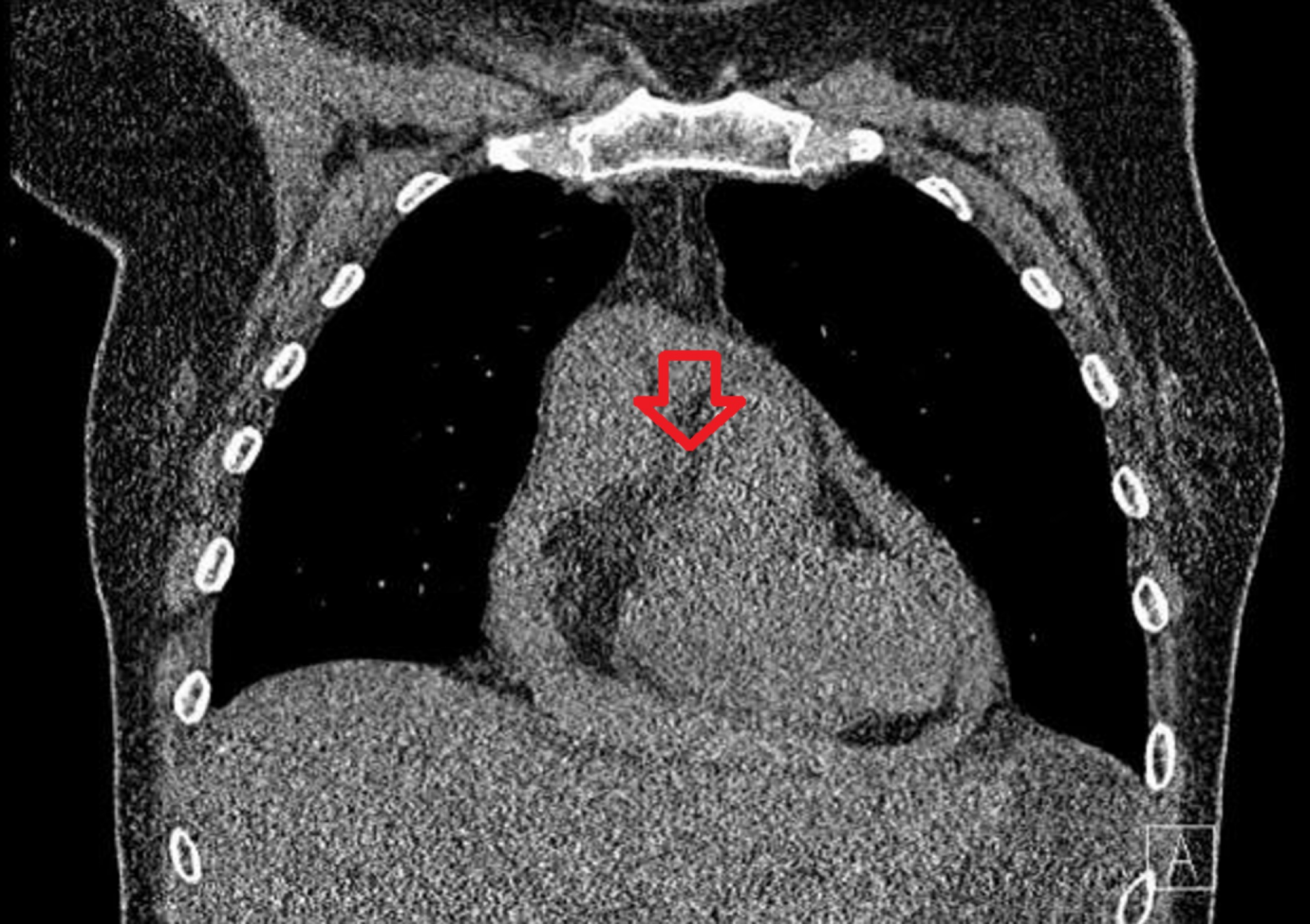
CT chest without contrast showed a large hemorrhagic appearing pericardial effusion and aneurysmal ectasia of the ascending aorta

Over the next few hours, the patient became hypotensive requiring ICU transfer for vasopressor support. The patient was scheduled for a pericardiocentesis but prior to the procedure, a computerized tomography (CT) chest without contrast showed a large hemorrhagic appearing pericardial effusion and aneurysmal ectasia of the ascending aorta (Figure 5).

**Figure 6:**
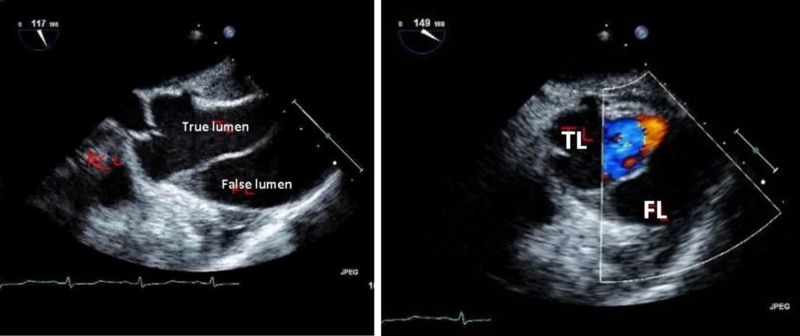
Transesophageal echocardiogram showing Stanford type A acute aortic dissection with true and false lumens

Emergent decision was made to call off the pericardiocentesis, and a transesophageal echocardiogram was performed showing a Stanford type A AAD beginning at the sinotubular junction with minimal aortic regurgitation (Figure 6).

**Figure 7:**
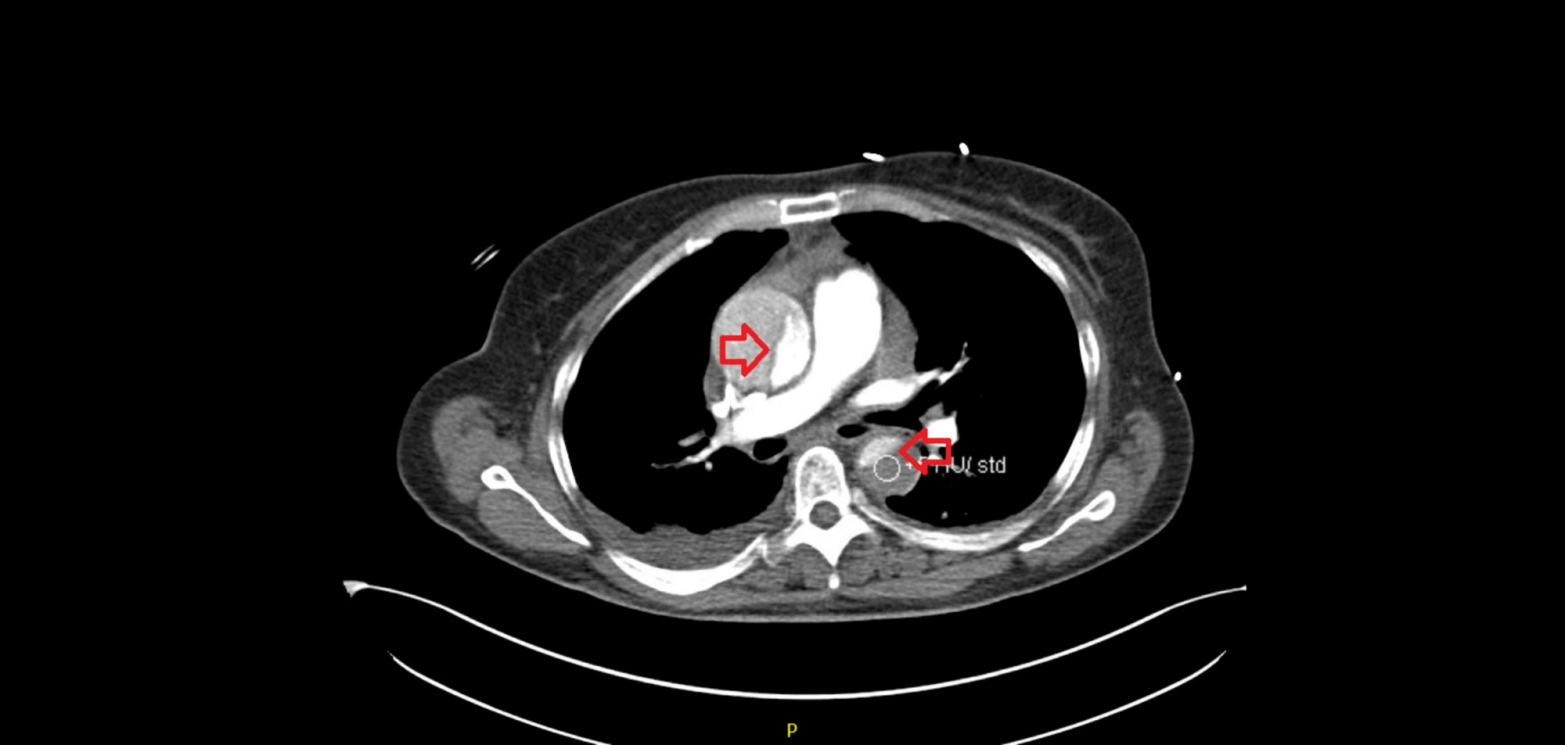
CT angiogram revealing Stanford type A AAD originating from the ascending and extending down the descending aorta

The patient had a CT angiogram revealing Stanford type A AAD extending into the arch and to the bifurcation (Figure 7).

The patient underwent elephant trunk repair of the ascending aorta with a favorable outcome.

## Discussion

The overall incidence of acute aortic syndromes ranges from two to four cases per 100,000 individuals [1]. AAD comprises the majority of acute aortic syndromes [5,6]. Risk factors associated with AAD include hypertension, atherosclerosis, prior cardiac surgery, known aneurysm, known connective tissue disorder, bicuspid aortic valve, and prior aortic surgery [7].

AAD presents with a wide range of manifestations, and classic findings are often absent. The clinical presentation of a dissection is that of the sudden onset of “tearing” chest pain that radiates to the back, with associated pulse or blood pressure deficit between upper limbs and an abnormal chest x-ray which is present in only one-third of patients [8]. It is important to rapidly distinguish ascending AAD, which is a cardiac surgical emergency, from descending thoracic aneurysm, which is managed medically in hemodynamically stable patients with no end organ damage. A thorough history is shown to be useful and a clinical examination is very important but is insufficiently sensitive to rule out aortic dissection given the high morbidity of a missed diagnosis. CT angiography is the diagnostic imaging modality of choice in hemodynamically stable patients. It is less operator dependent, provides useful anatomic correlates for surgical and endovascular therapy, and collects information for follow-up analysis and measurement (sensitivity of 83%-95% and specificity of 87%-100%) [9,10]. In hemodynamically unstable patients, transesophageal echocardiography is the initial study of choice, as it is a portable procedure that yields a diagnosis within minutes, with the addition of color-flow Doppler patterns that may decrease the false positives by recognizing differential flow velocities in the true and false lumens (Se 98% and Sp 65%-95%) [11]. The potential drawbacks of TEE include procedural sedation and need of experienced operators for accurate results.

AADs are frequently mistaken for other etiologies that cause chest pain, the most common being acute coronary syndromes. The incidence of initial miss diagnosis is up to 40% and may be more common when the ascending aorta is involved [12,13]. Errors in diagnosis delay proper treatment and can lead to inappropriate therapies (e.g., antithrombotic agents), which increases the risk of complications. This results in administration of aspirin and other antiplatelets and is associated with major bleeding and greater in-hospital mortality [2]. It also protends an increased risk of a hemorrhagic pericardial effusion, which we presume happened in case no. 2.

Tamponade resulting from pericardial effusion has been reported in 18.7% of the patients with Stanford type A AAD with worse outcomes [14]. The proposed mechanism is the transudation of fluid across the thin wall of the false aortic lumen into the pericardial space. More rarely, the dissected aorta ruptures directly into the pericardium, leading to rapid tamponade physiology. Urgent direct aortic repair together with intraoperative pericardial draining is the recommended treatment approach for these patients [15]. Preoperative controlled pericardiocentsis can be lifesaving when managing patients with critical cardiac tamponade complicating acute Stanford type A AAD, especially when cardiac surgery is not immediately available [16].

## Conclusions

AAD remains a diagnostic challenge for the physicians mainly due to its relatively low frequency and wide range of clinical presentations. It most often mimics acute coronary syndrome, subsequently leading to potentially hazardous management approaches and disastrous consequences. The definite diagnosis relies on imaging studies to define the aortic abnormality, classify the location and extent, and identify any anatomic complications. A crucial aspect of early therapy is ensuring a correct diagnosis so that an appropriate management scheme can be instituted in a timely fashion.
